# A case presentation of a metachronous superficial epiglottic lesion excised by endoscopic submucosal dissection

**DOI:** 10.1055/a-2725-7222

**Published:** 2025-11-19

**Authors:** Chenchen Zhang, Zhaosheng Chen, Nan Zhang, Daoyu Tao, Honglei Wu

**Affiliations:** 1 66555 Department of Gastrointestinal Endoscopy Center, The Second Qilu Hospital, The Second Clinical Medical School of Shandong University, Jinan, China; 2 66555 Department of Pathology, The Second Qilu Hospital, The Second Clinical Medical School of Shandong University, Jinan, China

A case presentation of a metachronous superficial epiglottic lesion excised by endoscopic submucosal dissection.

Previous reports 1 2 have shown that patients with esophageal squamous cell carcinoma (ESCC) have an increased risk of synchronous and metachronous squamous cell carcinoma of the head and neck. The piriform sinus and posterior pharyngeal wall are the most common sites for the development of metachronous lesions. Systematic endoscopic pharyngeal evaluation is vital for the surveillance of the regions 3 . Here, we present a metachronous superficial lesion in the vallecula epiglottica – a rare site – which occurred after superficial ESCC.

A 69-year-old man underwent endoscopic submucosal dissection (ESD) for circumferential superficial cancer in the upper thoracic esophagus 1.5 years ago. He was diagnosed with a superficial neoplastic lesion in the vallecula epiglottica during a follow-up gastroscopy recently. A 1.0-cm, slightly reddish with whitish plaques, flat (0–IIb), well-demarcated lesion was detected under white light ( Fig. 1 **a** ), demonstrating pale brown under narrowband imaging (NBI) ( Fig. 1 **b** ). Under magnifying endoscopy with NBI, type B1 vessels were detected in the reddish area, and the vessels were vague in the whitish area ( Fig. 1 **c, d** ). Biopsy results revealed carcinoma in situ, and a computed tomography scan revealed no metastasis. Under tracheal intubation anesthesia, the lesion was removed by ESD using a GOLDKNIFE (Micro-tech, Nanjing, China), and no adverse events occurred ( Video 1 ). The lesion was marked under NBI with ME distinctly, following a circumferential mucosal incision and submucosal dissection. En bloc resection of the lesion was performed in this anatomically challenging region. Histopathology confirmed carcinoma in situ with negative margins ( Fig. 2 ).

**Fig. 1:**
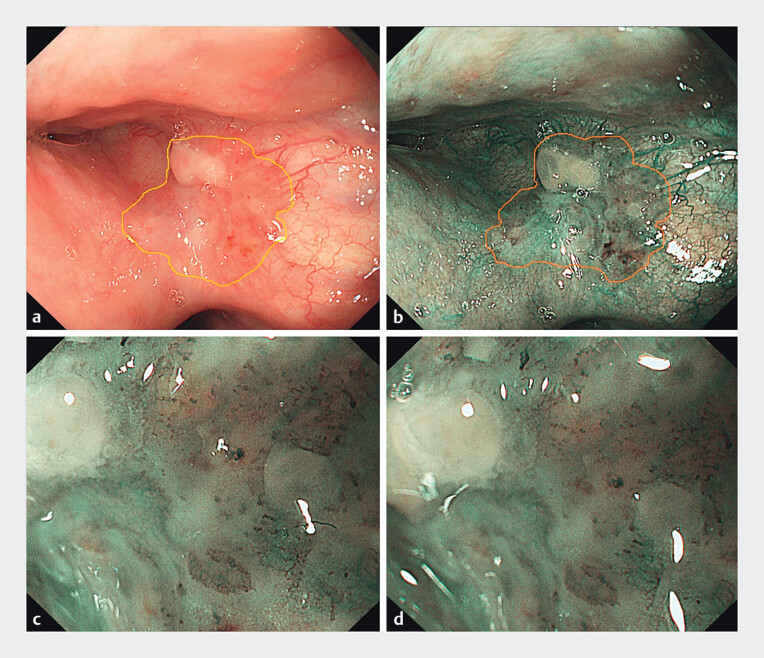
Endoscopic features of the lesion. **a** White light view: slightly reddish with whitish plaques, Paris 0-IIb, well-demarcated; **b** pale brown under narrowband imaging (NBI); and **c, d** magnifying endoscopy findings with NBI: type B1 vessels (JES) were detected in the reddish area, while the vessels appeared vague in the whitish area.

Gastroscopy showing endoscopic features of the lesion and the procedure of endoscopic submucosal dissection (ESD).Video 1

**Fig. 2:**
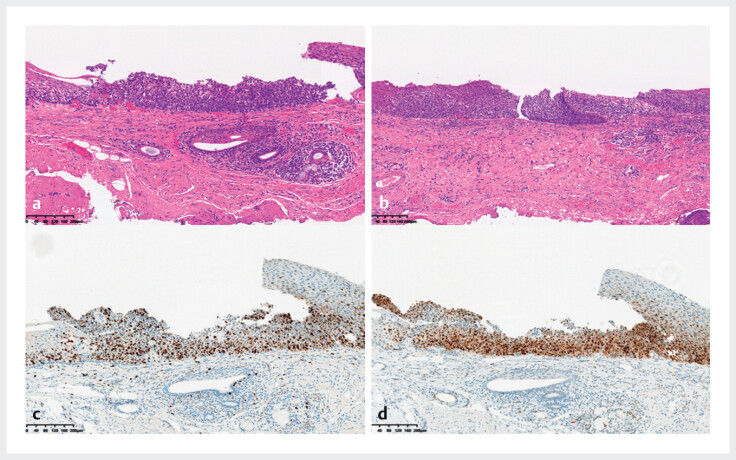
Histopathological findings of the endoscopically dissected specimen. **a, b** HE staining (10×); the tumor shows strong positivity for ( **c** ) Ki67 and ( **d** ) P53.

In conclusion, we present the rare case of early pharyngeal cancer in the epiglottic vallecula. Considering that ESD has been a feasible and effective treatment for early pharyngeal lesions, detailed endoscopic pharyngeal evaluation is vital for early detection, especially in patients with an ESCC history.

Endoscopy_UCTN_Code_CCL_1AB_2AB

## Notes

Endoscopy E-Videos https://eref.thieme.de/e-videos
*E-Videos* is an open access online section of the journal *Endoscopy* , reporting on interesting cases and new techniques in gastroenterological endoscopy. All papers include a high-quality video and are published with a Creative Commons CC-BY license. Endoscopy E-Videos qualify for HINARI discounts and waivers and eligibility is automatically checked during the submission process. We grant 100% waivers to articles whose corresponding authors are based in Group A countries and 50% waivers to those who are based in Group B countries as classified by Research4Life (see: https://www.research4life.org/access/eligibility/ ). This section has its own submission website at https://mc.manuscriptcentral.com/e-videos .
